# Glycaemic control in diabetic patients in Zambia

**DOI:** 10.11604/pamj.2014.19.354.5264

**Published:** 2014-12-05

**Authors:** Emmanuel Mwila Musenge, Alexey Manankov, Boyd Mudenda, Charles Michelo

**Affiliations:** 1 Department of Physiological Sciences, School of Medicine, University of Zambia, Lusaka, Zambia; 2 Department of Public Health, Section for Epidemiology and Biostatistics, School of Medicine, University of Zambia, Lusaka, Zambia

**Keywords:** Diabetes mellitus, glycaemic control status, glycosylated haemoglobin, fasting plasma glucose, Zambia

## Abstract

**Introduction:**

The glycaemic control status of diabetic patients affects the management of their disorder. We examined the glycaemic control and clinical factors that may influence the achievement of the glycaemic control targets among diabetic out-patients.

**Methods:**

This was a hospital based cross-sectional study carried out at the University Teaching Hospital diabetic clinic in Lusaka, Zambia. A simple random sample of 198 consenting participants was selected from diabetic out-patients between September and December 2013. A structured interview schedule was used to capture socio-demographic data as well as needed clinical data from clients’ medical records and laboratory results. Multivariate binary logistic regression analysis was carried out to examine factors that may be associated with the glycaemic control status of these diabetic patients.

**Results:**

Overall (n = 198), mean (SD) age was 53.19±13.32 years. Majority (61.3%) of the patients had poor glycaemic control status (HbA_1c_≥49 mmol/mol). Insulin treatment (OR 0.13, 95% CI: 0.01 - 1.41), systolic blood pressure (OR 1.04, CI: 1.00 - 1.08) and fasting plasma glucose (previous; OR 0.81, CI: 0.72 - 0.90 and current; OR 0.85, CI: 0.78 - 0.93) were statistically significantly associated with glycaemic control. The poor glycaemic control observed in this study is similar to that reported in other published studies.

**Conclusion:**

We found evidence of poor glycaemic control in the study population suggesting need to explore the reasons for this. Association of Insulin, systolic blood pressure and fasting plasma glucose with glycaemic control further suggests the efficiency of traditional basic monitoring parameters which should be exploited in sharpening primary preventive strategies especially those that support lifestyle modification. Such efforts should also be integrated in all information, education and communication strategies that target but not limited to hospital based patients too.

## Introduction

Diabetes mellitus (DM), a metabolic disorder of hyperglycaemia due to insulin deficiency, or insulin resistance or both [1] is one of the major causes of premature illness and death worldwide [2]. The worldwide prevalence of DM among adults (20-79 years) was 285 million (6.4%) in 2010 [2] and is projected to increase to 552 million (7.7%) by 2030 [3, 4]. The percentage of deaths attributable to DM in the whole world was 5.5% in 2010 [2]. In 2013, 382 million (8.3%) adults worldwide were living with DM and a further 316 had impaired glucose tolerance. Most of these were aged between 40 and 59 years [5, 6]. Further, this report indicates that 19.8 million of the 382 diabetics were adults living in sub-Saharan Africa which is nearly double the number previously estimated by Sicree et al. [7] 5 years earlier who had estimated a 12.1 million people in sub-Saharan Africa living with diabetes and had projected the number to rise to be over 23.9 million by 2030 [6, 8]. The sub-Saharan African adult DM prevalence was 2.4% [2]. These reports signify the continuing increase and it is estimated that the diabetic population in Africa will double from that of 2013 by 2035 [5].

In 2013, the report estimated that, half of the reported deaths from diabetes occurred in patients aged less than 60 years but in Africa over 76% deaths occurred in patients under 60 years (in their prime productive years) [5]. The increase can be explained by the adoption of a Western diet in place of more healthy “traditional” diets for those in the developing world, as well as adopting a more sedentary lifestyle which is ubiquitous in developed countries. That said, type 2 diabetes is increasingly becoming a major health concern in rural communities in low and middle-income countries suggesting no communities or countries will escape this epidemic [7].

Glycosylated haemoglobin (HbA_1c_) is a gold standard in analysis of patients’ glycaemic control status, and is essential to ensure the optimal care of diabetic patients [9]. It also serves as a marker for average glycaemic levels over the previous 8 to 12 weeks prior to the measurement [10] and may be used to monitor the effects of diet, exercise, and drug therapy on glycaemia in diabetic patients.

The control of DM has proved to be difficult among those already with the disease as they are unable to monitor and maintain near-normal glycaemic levels [11]. Some factors that influence glycaemic control include body mass index (BMI), adherence, diabetes duration, blood pressure and type of medication [12].

Several studies have been conducted among diabetic patients in developed and developing countries. In China, the proportion of patients with tight glycaemic control was 40.2% [13]. Age, duration, oral anti-diabetic drugs and DM education predicted these levels. The glycaemic control status in diabetic patients in Brazil was also poor at 76% and the factors significantly associated with the control status included shorter DM duration, multi-professional care, participation in a DM health education programme, and satisfaction with current DM treatment [14].

In Nigeria, about 64% of the patients had HbA_1c_ value greater than 55 mmol/mol [15]. Similarly, in Kenya 39.5% of the patients had mean HbA_1c_ < 64 mmol/mol, while 60.5% had HbAlc > 64 mmol/mol [16]. Diet and weight loss was associated with best control, because of possible fair endogenous increased insulin sensitivity. In contrast, good glycaemic control status was reported in Japan and Germany (45% and 65%), possibly because of the higher literacy levels with consequent probable better knowledge about DM [17, 18]. In Malawi, the overall prevalence of impaired fasting blood glucose (FBG) was 4.2% [8].

In Zambia, 8% of the studied population had hyperglycaemia with 3% DM prevalence in males and 4% in females [19]. However, this might be a huge under-estimation of the burden given the challenges associated in collecting and analysing this kind of data. Thus, the estimated mortality of patients with type 2 DM in 2009 in Zambia had doubled from 100 a year earlier [19]. These mortality rates may be greatly understated given that diabetes is frequently a component important of other non-communicable disease such as cardiovascular, renal with high co-morbidities and some deaths may be subscribed to other diseases still including pneumonia, tuberculosis, human immunodeficiency virus or sepsis when most of the contribution was from diabetes [19].

Nonetheless a needs assessment for diabetes treatment and countrywide management, carried out by the Zambian government for the Non-Communicable Disease programme, showed that there were inadequacies in terms of drugs and laboratory reagents; diagnostic facilities; expertise in disease management and community awareness for this disease [11].

However, health care workers and program managers in Zambia have not been using HbA_1c_ fully in glycaemic control monitoring especially in government health facilities. Most of the diabetic out-patients who visit the University Teaching Hospital (UTH), the main tertiary referral centre in the country, do not monitor their blood glucose levels at home as it is not feasible for them. The morbidity and mortality due to DM at the UTH was 561 (7.7%) and 114 (20.3%) respectively in 2010 [20]. There is thus paucity of data in Zambia on glycaemic control status in diabetic patients using HbA_1c_.

We examined the current glycaemic control outcomes, and identified the clinical factors that may influence glycaemic control in diabetic out-patients at the UTH in the Lusaka province of Zambia. It was hoped that this information would enable health care providers to improve and adjust where necessary the management of diabetic out-patients upon knowing their long-term glycaemic control status.

## Methods

### Population and sampling procedures

Data stem from a hospital based cross-sectional study carried out at the Diabetic Clinic run at the UTH, Lusaka, Zambia. The UTH is the national referral health centre that treats and reviews patients with various diseases, including DM. The patients attend the clinic at appointed times advised by the medical officers for continuous monitoring and consultation about their disease.

All the confirmed diabetic out-patients for at least two years and aged 15 years and above were included in the study. The patients who were willing to participate in the study were asked to give informed and written consent.

However, the newly diagnosed patients and those who were recruited in the previous month(s) were excluded from the study. A simple random sampling method was employed and patients were selected consecutively from September to December, 2013 to avoid sampling bias. The patients were selected based on the daily sampling frame.

The sample size was calculated basing on Krejcie and Morgan's [21] formula for calculating sample size of a finite population. The calculated sample size comprised 186 participants. However, a higher number of 198 patients were targeted in order to account for possible refusals or exclusions and the need to carry out subgroup analysis.

### Data collection

A structured interview schedule was used to capture data on demographic characteristics, clinical factors and laboratory measurement results. The interview schedule was developed based on the World Health Organization (WHO) stepwise survey (STEPS) instrument [22]. The same instruments were used on all the patients to ensure reliability and validity. The data on demographic, clinical factors, fasting plasma glucose (FPG) and HbA_1c_ were obtained by interview, review of medical records and anthropometric measurements.

The weight and height of the patients were measured using a ZT-160 adult weighing mechanical scale model with a height rod (Wuxi Weigher Factory Co., Ltd, Zhejiang, China) whose values were used to compute the body mass index BMI based on the formula developed by Lambert Adolphe Jacques Quételet in 1835 [23]. A scientific calculator FX-82ES (CASIO computer company Ltd, Tokyo, Japan) was used to obtain the actual BMI figure by dividing weight in kilograms with height squared in metres which was also verified by the WHO BMI chart [24]. The blood pressure was measured using the Citizen Digital Blood Pressure Monitor (Citizen Systems Japan Co., Ltd, Tokyo, Japan).

### Laboratory examination

The quantitative determination of HbA_1c_ level, a marker of DM, in the collected blood from the patients was carried out by the immunoturbidimetry method using the ABX Pentra 400 discrete photometric benchtop Automated Clinical Chemistry Analyser (HORIBA ABX SAS, 34184 Montpellier, France), whose technique has been certified by the National Glycohaemoglobin Standardisation Program (NGSP) of Australia [25]. The FPG was measured by the enzymatic determination of glucose using the Trinder method using the same analyser [26]. The therapeutic objective of HbA_1c_ has been to obtain values ≤ 48 mmol/mol as recommended by the International Diabetes Federation (IDF) and American College of Endocrinology (ACE) [27]. The target for FPG is ≤ 6 mmol/L.

### Data analyses

Statistical analyses were carried out using IBM^®^ SPSS^®^ Statistics for Windows Version 20.0 (IBM Corp. Armonk, NY, USA). The frequencies and descriptive statistics of the variables were calculated. The Chi-squared, Fisher's exact and students’ t-tests were used to select potential predictors of good or poor glycaemic control status. The Odds Ratio and 95% confidence interval were calculated using binary logistic regression to identify predictors of glycaemic control while adjusting for confounders. A p-value of < 0.05 was considered significant.

### Ethics approval

This study was approved by the University of Zambia Biomedical Research Ethics Committee (Reference number 005-07-13).

## Results

### Participation and distribution

Of the 198 patients included in the study, 119 (60.1%) were females and the median age was 55 years (IQR±17). Fewer than half 92 (46.5%) of the patients had secondary education.

### Clinical factors and laboratory measurement results

The majority (92.9%) of the patients had type 2 DM and 79.8% of the patients had been diabetic for 2-10 years while only three (1.5%) had been diabetic for 21 years and above. More than half (56.6%) of the patients were on oral anti-diabetic drugs and seven (3.5%) were on both oral anti-diabetic drugs and insulin. Most (130; 65.7%) of the participants were on non-antidiabetic treatment and 34.3% were on none. In addition, 64.6% of the patients had co-morbidity while 35.4% had none. Fewer than half (70; 36.8%) of the patients with complete data were overweight (25-29.9 kg/m^2^) and six (3%) were underweight (< 18.4 kg/m^2^) as per the WHO classification of obesity. Also, more than half (117; 59.1%) of the patients reported history of DM in the family while 79 (40.9%) reported none. The mean (SD) systolic blood pressure (SBP) and diastolic blood pressure (DBP) were 132.7 ± 17.90 mm Hg and 84.7 ±11.18 mm Hg.

**Figure 1:**
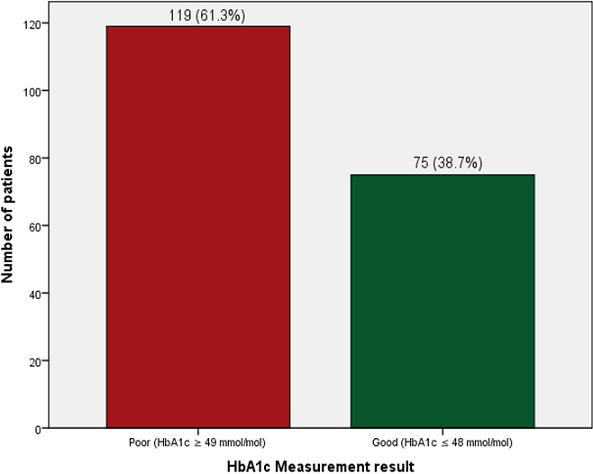
HbA1c measurements results

The mean (SD) FPG of the patients for the past three months and current FPG were 10.71 ± 7.75 mmol/L and 10.98 ± 6.22 mmol/L. Only (75; 38.7%) of the patients had good glycaemic control status (HbA_1c_ ≤ 48 mmol/mol) while most (119; 61.3%) of the patients had poor glycaemic control status (HbA_1c_ ≥ 49 mmol/mol) among those whose data were complete (Figure 1).

### Factors associated with glycaemic control status

The Chi-square, Fisher's exact and students’ t-tests were run to select the variables to include into the regression model. The distribution of glycaemic control status of the patients by the demographic and clinical factors is shown in Table 1 and Table 2. There was an association between glycaemic control status and age, type of anti-diabetic treatment, non-antidiabetic treatment, co-morbidity, SBP, previous and current FPG. Nonetheless, sex, education, type of DM, duration of DM, BMI, DBP and family history of DM, were not associated with glycaemic control status (Table 1 and Table 2).

**Table 1:** Glycaemic control status by demographic factors of patients

	Glycaemic control status		
	Good (n = 75, HbA1c ≤ 48 mmol/mol)	Poor (n = 119, HbA1c ≥ 49 mmol/mol)	
Characteristic	No (%)	No (%)	*P*-value*
**Age**			**0.018**^**a**^
15-34 years	3 (12.5)	21 (87.5)	
35-54 years	29 (40.8)	42 (59.2)	
55 years and above	43 (43.4)	56 (56.6)	
**Sex**			0 .908^a^
Male	29 (38.2)	47 (61.8)	
Female	46 (39.0)	72 (61.0)	
**Education level**			0.053^a^
Never/Primary	27 (36.5)	47 (63.5)	
Secondary	33 (37.5)	55 (62.5)	
College/University	15 (46.9)	17 (53.1)	

* *P* < 0.05.

a Pearson's Chi-squared test

**Table 2:** Glycaemic control status by clinical factors of the patients

	Glycaemic control status		
	Good (n = 75, HbA1c ≤ 48 mmol/mol)	Poor (n = 119, HbA1c ≥ 69 mmol/mol)	
Characteristic	No (%)	No (%)	P-value*
**DM type**			0.052^a^
Type 1	2 (14.3)	12 (85.7)	
Type 2	73 (40.6)	107 (59.4)	
**DM duration**			0.160^a^
2-10 years	65 (41.9)	90 (58.1)	
11-20 years	9 (25.0)	27 (75.0)	
21 years and above	1 (33.3)	2 (66.7)	
**DM treatment type**			**0.030** ^**b**^
Diet/none	6 (66.7)	3 (33.3)	
Oral antidiabetic drugs and Insulin	2 (28.6)	5 (71.4)	
Insulin	18 (26.9)	49 (73.1)	
Oral antidiabetic drugs	49 (44.1)	62 (55.9)	
**Non-DM treatment drugs**			**0.032** ^**a**^
No	19 (28.4)	48 (71.6)	
Yes	56 (44.1)	71 (55.9)	
**Co-morbidity**			**0.040** ^**a**^
No	20 (29.0)	49 (71.0)	
Yes	55 (44.0)	70 (56.0)	
**Body Mass Index (kg/m2)**			0.178^b^
Underweight (≤18.4)/Normal (18.5-24.9)	18 (29.0)	44 (71.0)	
Overweight (25-29.9)	31 (44.3)	39 (55.7)	
Obese (≥ 30)	22 (40.7)	32 (59.3)	
**SBP (mm Hg; Mean, SD)**	130.64 (18.45)	136.40 (16.49)	**0.029** ^**c**^
**DBP (mm HG; Mean, SD)**	86.24 (9.91)	84.01 (11.80)	0.175^c^
**DM in Family**			0.161^a^
No	36 (44.4)	45 (55.6)	
Yes	39 (34.5)	74 (65.5)	
**Previous FPG (mmol/L; Mean, SD)**	8.15 (3.42)	11.64 (5.58)	**0.000** ^**c**^
**Current FPG (mmol/L; Mean, SD)**	8.49 (4.63)	12.25 (6.09)	**0.000** ^**c**^

* *P* < 0.05.

a Pearson's Chi-Squared Test

b Fisher's Exact Test

c Students’ t-test

The multivariate binary logistic regression model was tested for multicollinearity, Hosmer and Lemeshow test of model fitness for data, omnibus test of model coefficients and classification accuracy. The dependent variable was glycaemic control status: Good (1), Poor (0). The results of the multivariate binary logistic regression analysis to predict whether nine variable factors, namely, age, education level, type of DM, type of DM treatment, non-antidiabetic treatment, comorbidity, SBP, previous and current FPG were associated with glycaemic control status showed that type of DM treatment, SBP, previous and current FPG, were statistically significantly associated with glycaemic control (Table 3).

**Table 3:** Multivariate binary logistic regression model-determining factors associated with glycaemic control status

Glycaemic control status				
	Good (n = 75, HbA1c ≤ 48 mmol/mol)	Poor (n = 119, HbA1c ≥ 49 mmol/mol)		
Predictor variable	No (%)	No (%)	AOR (95% CI)^a^	P-value*
**Age**				
15-34 years	3 (12.5)	21 (87.5)	0.34 (0.07-1.71)	0.192
35-54 years	29 (40.8)	42 (59.2)	1.04 (0.50-2.12)	0.922
55 and above	43 (43.4)	56 (56.6)	Ref (1.00)	
**DM type**				
Type 1	2 (14.3)	12 (85.7)	0.80 (0.13-4.82)	0.808
Type 2	73 (40.6)	107 (59.4)	Ref (1.00)	
**Treatment adherence**				
No	61 (38.1)	99 (61.9)	0.38 (0.13-1.07)	**0.043**
Yes	14 (41.2)	20 (58.8)	Ref (1.00)	
**DM treatment type**				
Oral antidiabetic drugs	49 (44.0)	62 (55.9)	0.20 (0.2-1.85)	0.154
Insulin	18 (26.9)	49 (73.1)	0.13 (0.01-1.41)	**0.044**
Oral antidiabetic drugs and insulin	2 (18.6)	5 (71.4)	0.50 (0.03-8.86)	0.640
Diet/None	6 (66.6)	3 (33.3)	Ref (1.00)	
**Non-DM treatment type**				
No	19 (28.4)	48 (71.6)	0.95 (0.26-3.46)	0.940
Yes	56 (44.1)	71 (55.9)	Ref (1.00)	
**Co-morbidity**				
No	20 (29.0)	49 (71.0)	0.74 (0.21-2.63)	0.638
Yes	55 (44.0)	70 (56.0)	Ref (1.00)	
**SBP (mm Hg; Mean, SD)** ^**a**^	130.64 (18.44)	136.40 (10.49)	1.04 (1.00-1.08	**0.038**
**Previous FPG (mmol/L; Mean, SD)**	8.15 (3.42)	11.64 (5.58)	0.81 (0.72-0.90)	**0.000**
**Current FPG (mmol/L; Mean, SD)**	8.49 (4.63)	12.25 (6.09)	0.85 (0.78-0.93)	**0.001**

* Indicates significant *p* **-**value at *p* < 0.05.

a AOR = adjusted odds ratio when other predictor variables were controlled for

The patients who were on insulin treatment tended to be less likely to achieve good glycaemic control status compared to those who were on diet/none although this was statistically significant. Each 1-mm Hg increase in SBP resulted in a 5% (OR 1.05, 95% CI: 1.00 - 1.10) increase in odds of having poor glycaemic control status. Moreover, the patients who had mean (SD) previous FPG, 11.64 ± 5.58 mmol/L and current FPG 12.25 ± 6.09 mmol/L were 16% and 15% (OR 0.81, 95% CI: 0.72 - 0.90 and OR 0.85, 95% CI: 0.78 - 0.93) less likely to achieve good glycaemic control status (Table 3).

## Discussion

In 2009 the WHO recommended that HbA_1c_ could be used as a diagnostic test for diabetes provided stringent quality control assurance tests were in place and assays were standardised to criteria aligned to the international reference values and there were no conditions which precluded its accurate measurements [28]. In addition they recommended that HbA_1c_ of lower than 48 mmol/mol did not exclude diagnosis of DM [29]. When used for diagnosis of type 2 DM the HbA_1c_ results determination are as follows: HbA_1c_ of < 39 mmol/mol means no DM (i.e. normal); when between 39 mmol/mol and 47 mmol/mol a diagnosis of pre-diabetic condition is achieved and DM as a diagnosis is considered when HbA_1c_ is 48 mmol/mol or higher with a firm diagnosis if greater 53 mmol/mol [30].

Individuals with HbA_1c_ persistently higher than 53 mmol/mol are at greater risk of developing eye, cardiac, and renal diseases as well as nerve damage and are at risk of suffering strokes and/or myocardial infarction [31]. While these definitions are limited by the source of data from which they were derived, these cut-off points and definitions have been shown to differentiate individuals with significantly increased premature mortality, and increased microvascular and/or cardiovascular complications. Further the WHO recommends that the lower limit of normal fasting glucose level of 6.1mmol/L be maintained [29].

However, the International Committee on diabetes, WHO and the American Diabetic Association (ADA) all agree that patients with HbA_1c_ levels of between 39-47 mmol/mol are at high risk diabetic complications and be considered pre-diabetics based on a pooled analysis of 43,364 people HbA_1c_ of 40 to 56 mmol/mol which was associated with retinopathy the primary factor used in determining the cutoff points of DM by FBG and 2-hour glucose tolerance test [32]. Thus using the HbA_1c_ alone could be missing out a large number of patients at high risk for DM complications. Consequently many more individual with diabetes (beyond those with suspected of poor control) in this study are at risk of diabetic complications.

In Zambia, the poor glycaemic control status in diabetic patients, coupled with an increase in the prevalence of DM, is a public health concern. Resources are being provided for the management of diabetic patients at both personal and government levels but achieving good glycaemic control status is proving to be a considerable challenge in most cases.

The current study showed that glycaemic control in most diabetic out-patients at the UTH was poor. Poor glycaemic control status has also been reported in other studies in developing and developed countries. The possible explanation for this state of affairs might be inadequate knowledge on treatment protocols, inactivity and poor diet among things. On the other hand, good glycaemic control status was reported in Japan and Germany [17, 18]. The good glycaemia control status in these countries might be because of the higher literacy levels, probably resulting in better knowledge about DM.

The four variables found to predict glycaemic control status in the current study were type of anti-diabetic treatment, SBP, and previous and current FPG. This study revealed that the patients on insulin were less likely to achieve good glycaemic control compared to those who were on diet/none.

These results agree with the findings by Ahmad, Islahudin and Paraidathathu where patients receiving insulin treatment had the highest mean HbA_1c_ level compared with those receiving monotherapy or a combination of oral antidiabetic drugs [33]. Thus, it appears as if control among insulin users tends to be more difficult because these patients have a more severe form of the disease or are in the late stages of the disease. Also, the procedure of insulin injection administration and the resources required for this procedure probably affect adherence. This calls for aggressive treatment and monitoring, both in terms of adequate dosing and improved adherence, to achieve better outcomes. In addition, studies have shown that intensive insulin therapy alone in type 2 DM patients results in excellent glycaemic control [34].

However, a study in Iran reported good glycaemic control among insulin users but the results were not statistically significant [35]. In another study, intensive insulin therapy alone in type 2 DM patients reported excellent glycaemic control [34]. Chuang et al. also reported that, the use of insulin or a combination of oral antidiabetic drugs was associated with improved glycaemic control [36]. The good glycaemic control among non-insulin users could be due to a more simple to administer treatment option, which tends to be more effective under the conditions of daily life.

Furthermore, the current study showed that an increase in SBP reduced the likelihood of achieving good glycaemic control status. Similarly, significant association between SBP and achievement of good glycaemic control status was observed in other studies reviewed. In the UK, poor glycaemic control status in patients with poorly controlled blood pressure (BP) was reported [37]. In addition, other studies have shown that lowering BP to a mean of 144/82 mmHg significantly reduces the incidence of stroke, diabetes-related death, heart failure, microvascular complications, and visual loss. However, Ghazanfari et al. [38] in Iran reported a lower mean (SD) SBP for those who achieved good glycaemic control status of 119.67 ±17.63 mm Hg and 122.07 ± 16.33 mmHg for poor glycaemic control status, but the results were not statistically significant. This implies that, regular BP check together with glycaemic monitoring to a target of 130/85 mm Hg is recommended for diabetic patients [9]. Thus, more attention should be addressed to these primary preventative factors in the management of DM patients as they affect glycaemic control greatly.

This study has also shown that, the patients who had mean (SD) previous FPG, 11.64 ± 5.58 mmol/L and current FPG 12.25 ± 6.09 mmol/L were less likely to achieve good glycaemic control status. The results are better than those reported in the USA where the older adults with both impaired fasting glucose (IFG) and elevated HbA_1c_ had substantially increased odds of developing diabetes over seven years [39]. On the other hand, in Japan, a 0.5% increase in HbA_1c_ per 0.56 mmol/L increase in FPG adjusted for age, sex, BMI and family history of diabetes was reported [40]. The combined use of FPG and HbA_1c_ levels predicts the progression to diabetes in individuals with no apparent risk. In particular, the combination is recommended for individuals with a FPG ≥ 5.55 mmol/L. Therefore, the addition of elevated HbA_1c_ to the model with IFG results in improved discrimination and calibration. This may improve the management of diabetic patients with consequent improvement in their quality of life.

Several studies have shown that HbA_1c_ is an index of average glucose (AG) over the preceding weeks-to-months and there is a very predictable relationship between HbA_1c_ and AG. Understanding this relationship can help patients with diabetes and their health care providers to set day-to-day targets for AG based on HbA_1c_ goals. Also, FPG should be used with caution as a surrogate measure of AG and it is important to remember that HbA_1c_ is a weighted average of glucose levels during the preceding four months. Unless the patient's glucose levels are very stable month after month, quarterly and half-yearly measurement is needed to ensure that a patient′s glycaemic control remains within the target range.

It is possible that our estimates could have been affected by many confounding factors. Possible confounding factors, such as diet, anti-diabetic drugs, comorbidity, non-antidiabetic drugs and SBGM sub-variable quantification were neither measured nor analysed but will be the next focus in the phase that will follow. The other limitation was that the time between the previous estimation of FPG and the current FPG was not the same for all patients. Some patients had their previous FPG reading taken four to six months prior to the study.

In addition, since the study was a cross-sectional study, it is difficult to establish a “causal” relation between HbA_1c_ and the clinical factors. Furthermore, this study was carried out on a finite study population illustrated by the fact that only the participants who visited the UTH diabetic clinic during the period of data collection were included in the sample. On the other hand, we are also aware that since the data on medical records of the diabetic patients at the clinic were incomplete; it was difficult to follow the morbidity patterns. Lastly but not the least, another issue that stands out as a critical factor was the cost containment, especially of laboratory materials and supplies.

## Conclusion

The majority of the patients in the current study had poor glycaemic control status that was influenced by insulin, SBP and FPG. This state of affairs raises concerns on the adequacy of the care provided to diabetic out-patients at the UTH. Since diabetes like most other chronic diseases is progressive, the findings suggest that if the factors influencing glycaemic control are not adequately addressed, complications increase and drug therapy becomes much more complex with time. The study will enable health care providers to review their management of diabetic patients to ensure good glycaemic control. In order to achieve this, it is suggested that further research should be conducted to evaluate the role of the diabetic patients in the management of their diabetes, especially by strict limitation of carbohydrate intake, as this accounts for modifications such as weight control, exercise and diet.

## References

[1] Gardner G David, Shoback Dolores (2011). Greenspan's basic and clinical endocrinology.

[2] World Health Organization (2010). Preventing chronic diseases: A vital investment.

[3] Shaw JE, Sicree RA, Zimmet PZ (2010). Global estimates of the prevalence of diabetes for 2010 and 2030. Diabetes Res Clin Pract..

[4] Whiting DR, Guariguata L, Weil C, Shaw J (2010). International Diabetes Federation diabetes atlas: global estimates of the prevalence of diabetes from 2011 and 2030. Diabetes Res Clin Pract..

[5] International Diabetes Federation (2013). International Diabetes Federation Atlas.

[6] Schmidt Inês Maria, Hoffmann F Juliana, de Fátima Diniz Sander Maria, Lotufo A Paulo, Griep Härter Rosane, Bensenor M Isabela (2014). High prevalence of diabetes and intermediate hyperglycemia: The Brazilian Longitudinal Study of Adult Health (ELSA-Brasil). Diabetology & Metabolic Syndrome..

[7] Sicree Richard, Shaw Jonathan, Zimmert Paul (2009). The Global Burden: Diabetes and Impaired Glucose Tolerance.

[8] Msyamboza Kelias Phiri, Mvula J Chimwemwe, Kathyola Damson (2014). Prevalence and correlates of diabetes mellitus in Malawi: Population-based national NCD STEPS survey. BMC Endocrine Disorders..

[9] American Diabetes Association (2010). Standards of medical care in diabetes. Diabetes Care.

[10] Roszyk L, Faye B, Sapin V, Somda F, Tauveron I (2007). Glycated haemoglobin (HbA1c): today and tomorrow. Ann Endocrinol (Paris)..

[11] Zambia National Assembly http://www.parliamnet.gov.zm/index.php.

[12] Hartz A, Kent S, James P, Xu Y, Kelly M, Daly J (2006). Factors that influence improvement for patients with poorly controlled type 2 diabetes. Diabetes Res Clin Pract.

[13] Bi Y, Zhu D, Cheng J, Zhu Y, Xu N, Cui S, Li W, Cheng X, Wang F, Hu Y, Shen S, Weng J (2010). The status of glycemic control: A cross-sectional study of outpatients with type 2 diabetes mellitus across primary, secondary, and tertiary hospitals in the Jiangsu province of China. Clin Ther.

[14] Mendes Beatriz Valverde Ana, Fittipaldi Antônio Saraiva João, Neves Celestino Silva Raimundo, Chacra Roberto Antônio, Moreira Duarte Edson (2010). Prevalence and correlates of inadequate glycaemic control: results from a nationwide survey in 6,671 adults with diabetes in Brazil. Acta Diabetologica..

[15] Adebisi SA (2009). Glycated haemoglobin and glycaemic control of diabetics in Ilorin. Nigerian J Clin Pract..

[16] Otieno CF, Kariuki M, Ng'ang'a L (2003). Quality of glycaemic control in ambulatory diabetics at the out-patient clinic of Kenyatta National Hospital. East African Medical Journal..

[17] Reisig V, Reitmeir P, Döring A, Rathmann W, Mielck A (2007). Social inequalities and outcomes in type 2 diabetes in the German region of Augsburg: a cross-sectional survey. Int J Public Health..

[18] Arai Keiko, Hirao Koich, Matsuba Ikuro, Takai Masahiko, Matoba Kiyokazu, Takeda Hiroshi (2009). The status of glycemic control by general practitioners and specialists for diabetes in Japan: a cross-sectional survey of 15,652 patients with diabetes mellitus. Diabetes Res Clin Pract.

[19] Ministry of Health (2011). National Health Strategic Plan (NHSP) 2011-2015: Towards attainment of health related Millennium Development Goals.

[20] University Teaching Hospital (2010). University Teaching Hospital Action Plan 2009/2010: Health Management Information System.

[21] Krejcie RV, Morgan DW (1970). Determining sample size for research activities. Educ Psychol Meas..

[22] World Health Organization (2007). World Health Organization STEPwise approach to chronic disease risk factor surveillance (STEPS) instrument.

[23] Garabed Ehnoyan (2007). “Adolphe Quetelet (1796-1874) - The average man and indices of obesity.”. Nephrology Dialysis Transplantation.

[24] World Health Organization (2006). “BMI Classification” Global data base on BMI.

[25] Burtis CA, Ashood ER, Burns DE, Sacks D. B (2006). Carbohydrates: TIETZ Textbook of Clinical Chemistry and Molecular Diagnostics.

[26] Arvind Kumar, Rajiv Kr. Mishra, Sudhanshu S Roy (2004). “Studies on Impact of Industrial Pollution on Biochemical and Histological Changes in a Catfish, Mystus vittatus (Bloch)”.

[27] International Diabetes Federation (2009). International Diabetes Federation Atlas.

[28] World Health Organisation (2009). Use of Glycated haemoglobin (HbA1c) in the diagnosis of diabetes mellitus: Abbreviated report of a WHO consultation.

[29] World Health Organization (2006). Definition and diagnosis of diabetes mellitus and intermediate hyperglycaemia: Report of WHO/IDF consultation.

[30] American Diabetes Association (2011). Standards of medical care in diabetes. Diabetes Care.

[31] Cummins J (2013). Underwriting the applicant with diabetes: How important is glycaemic control?. Gne Re Risk Matters Oceania.

[32] World Health Organisation (2011). Use of glycated haemoglobin (HbA1c) in diagnosis of diabete s mellitus: Abbreviated report of WHO consultation.

[33] DeFronzo RA (1999). Pharmacologic therapy for type 2 diabetes mellitus. Ann Intern Med..

[34] Ghazanfari Zeinab, Niknami Shamsaddin, Ghofranipour Fazlollah, Larijani Bagher, Agha-Alinejad Hamid, Montazeri Ali (2010). Determinants of glycemic control in female diabetic patients: a study from Iran. Lipids in Health and Disease.

[35] Sufiza Ahmad Nur, Islahudin Farida, Paraidathathu Thomas (2013). Factors associated with good glycemic control among patients with type 2 diabetes mellitus. Journal of Diabetes Investigation.

[36] Chuang LM, Tsai ST, Huang BY, Tai TY (2002). The status of diabetes control in Asia - A cross-sectional survey of 24317 patients with diabetes mellitus in 1998. Diabet Med.

[37] Higgins GT, Khan J, Pearce IA (2007). Glycaemic control and control of risk factors in diabetes patients in an ophthalmology clinic: what lessons have we learned from the UKPDS and DCCT studies?. Acta Ophthalmol Scand..

[38] Bevan L Jeffrey (2010). Diabetes mellitus: A review of select ADA standards for 2006. J Nurse Pract..

[39] Lipska Kasia J, Inzucchi Silvio E, Van ness Peter H, Gill Thomas M, Kanaya Alka (2013). Elevated hba1c and fasting plasma glucose in predicting diabetes incidence among older adults. Diabetes Care.

[40] Inoue K, Matsumoto M, Kobayashi Y (2017). The combination of fasting plasma glucose and glycosylated hemoglobin predicts type 2 diabetes in Japanese workers. Diabetes Res Clin Pract..

